# Is the co-location of GPs in primary care centres associated with a higher patient satisfaction? Evidence from a population survey in Italy

**DOI:** 10.1186/s12913-017-2187-2

**Published:** 2017-04-04

**Authors:** Manila Bonciani, Sara Barsanti, Anna Maria Murante

**Affiliations:** Laboratorio Management e Sanità, Institute of Management, Scuola Superiore Sant’Anna, Pisa, Italy

**Keywords:** Primary care centre, Co-location, Multidisciplinary team, Patient satisfaction, Health care evaluation, Integrated care

## Abstract

**Background:**

Several countries have co-located General Practitioners (GPs) in Primary Care Centres (PCCs) with other health and social care professionals in order to improve integrated care. It is not clear whether the co-location of a multidisciplinary team actually facilitates a positive patient experience concerning GP care. The aim of this study was to verify whether the co-location of GPs in PCCs is associated positively with patient satisfaction with their GP when patients have experience of a multidisciplinary team. We also investigated whether patients who frequently use health services, due to their complex needs, benefitted the most from the co-location of a multidisciplinary team.

**Methods:**

The study used data from a population survey carried out in Tuscany (central Italy) at the beginning of 2015 to evaluate the patients’ experience and satisfaction with their GPs. Multilevel linear regression models were implemented to verify the relationship between patient satisfaction and co-location. This key explanatory variable was measured by considering both the list of GPs working in PCCs and the answers of surveyed patients who had experienced the co-location of their GP in a multidisciplinary team. We also explored the effect modification on patient satisfaction due to the use of hospitalisation, access to emergency departments and visits with specialists, by performing the multilevel modelling on two strata of patient data: frequent and non-frequent health service users.

**Results:**

A sample of 2025 GP patients were included in the study, 757 of which were patients of GPs working in a PCC. Patient satisfaction with their GP was generally positive. Results showed that having a GP working within a PCC and the experience of the co-located multidisciplinary team were associated with a higher satisfaction (*p* < 0.01). For non-frequent users of health services on the other hand, the co-location of multidisciplinary team in PCCs was not significantly associated with patient satisfaction, whereas for frequent users, the strength of relationships identified in the overall model increased (*p* < 0.01).

**Conclusion:**

The co-location of GPs with other professionals and their joint working as experienced in PCCs seems to represent a greater benefit for patients, especially for those with complex needs who use primary care, hospitals, emergency care and specialized care frequently.

## Background

The co-location of services and professionals within Primary Care Centres (PCCs) has been adopted in several countries as an organisational solution to improve integrated care. At the European level, co-location exists in Finland, France, Italy, Netherlands, Portugal, Spain, Sweden and the UK [1–4]. Beyond the existing differences in their organisational models, the common characteristic of these experiences is the co-location of General Practitioners (GPs) with other health and social care professionals in order to provide a more coordinated solution to patient needs.

A key feature of PCCs is the presence of multidisciplinary staff, although with different levels of inter-professional collaboration. In these settings, GPs play the role of a key point of contact for patients and their families with the overall health system, and GPs’ engagement within multidisciplinary teams may contribute to increased coherence in the patient care pathway. Indeed, the collaboration between GPs, nurses and social workers, as well as specialists form secondary care services, may benefit patients and their families in terms of coordination and continuity of care [5, 6], as well as in terms of a more efficient use of resources that allows to better balance the physician and non-physician labour input in the care process [7].

The multidisciplinary team approach has been recognised as playing an increasing role in the management of patient care [8–10]. This is particularly important for elderly patients [11] and in general for people with chronic conditions [12, 13], as well as for mental disorders [14] and cancer [15–17], or in acute conditions such as stroke rehabilitation [18]. Because of their complex needs, all these patients use health services frequently, and interact frequently with social and health professionals and in particular with different primary and secondary care providers, with a high risk of experiencing fragmentation in their care pathway [19]. The co-location of multidisciplinary teams in PCCs, which facilitates patients thanks to the concentration of different services in one facility with a single point of access, can improve the integration of patient care, ensuring a multi-perspective interpretation of patients’ needs, enabling professionals to share decisions on care and providing comprehensive and personalised care [20, 21]. Although multidisciplinary teams may also involve professionals from different organisations who work as a unique team without sharing the same practice, their co-location could represent a surplus value because it contributes to use time and resources more effectively, facilitate communication and information sharing, improve relationships between professionals and between professionals and parents [22].

Some authors have pointed out that co-location has a positive influence on processes and outcome of care [23–25], because it enables professionals to share information on patients and to jointly define their care pathways [26, 27]. The physical proximity of different providers in PCCs thus seems to make it easier to assume a person-focused approach in healthcare, especially when it also involves social and health integration [28].

However, there is no unanimous consensus regarding the relationship between co-location and a multidisciplinary approach in the primary care setting [9]. Some authors have doubted that the targets of multidisciplinary working can be simply achieved by pushing different professions together under the same roof [29, 30]. These authors have highlighted that co-located services have not changed the approach to healthcare provision: often they still operate as silo providers and co-location does not automatically lead to a multidisciplinary team [31]. In this sense, the patients of GPs working with other professionals in a co-located setting do not necessarily have experience of a multidisciplinary approach in their process of care. These assumptions are in contrast with the evidence that when professionals are co-located in the same structure, they are more likely to work jointly and collaborate reciprocally, thus achieving better results for users and an improvement in service quality [32–34].

To the best of our knowledge, no studies have been carried out yet to evaluate whether this co-location actually facilitates a positive patient experience concerning GP care.

Generally, the literature acknowledges the role of patient satisfaction in measuring the quality of health care [35–38] and shows broad evidence on factors associated with the patients’ perception of the quality of GP care and the need to know more about their determinants and components in order to improve the quality of care. Firstly, patient satisfaction has been found to be related to patient socio-demographic characteristics [39–42] and health conditions [42]. Other factors are related to the GP profile [39–41, 43], as well as the characteristics of general practice, such as the practice size [39, 40], personal list system [39, 40] and practice type [44]. The features of the national health system also influence patient satisfaction [45–47].

**Fig. 1:**
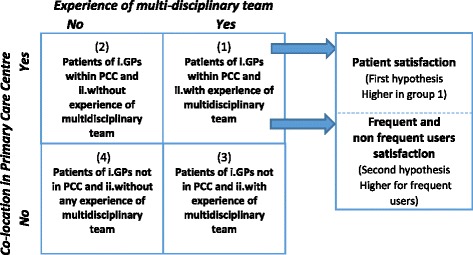
Conceptual model

Based on the above evidence, the aim of this study was to verify whether the co-location of GPs in PCCs is associated positively with patient satisfaction with the GP when their patients have experience of multidisciplinary team (Fig. 1).

In particular, our first hypothesis was that patients would be more satisfied when GPs are co-located with other health and social professionals in PCCs and, at the same time, they perceived their GPS and other professionals working as a multidisciplinary team. In fact, this group should evaluate better the care received from their GP compared to the other patients. Better of course than patients of GPs working or not in PCC who do not perceive the same multidisciplinary approach, but also than patients having experience of this approach but whose GPs work in different settings, such as policlinic or shared practices, which are not based on the same integrated care principles of PCCs.

Our second hypothesis was that this positive relationship mostly exists for patients who used health services frequently, since they should benefit more from the co-location of these multidisciplinary teams due to their complex needs.

These hypotheses were tested in Tuscany (central Italy).

## Methods

### Study setting

The Italian public health care system is inspired by the Beveridge model and it is characterized by public taxation funding, free access at the point of delivery (with some co-payments for specific visits) and healthcare system regionally managed. GPs are involved in delivering various primary care services, such as health promotion and preventive care activities, diagnosis, treatment, and the follow up of non-complex, acute, and chronic conditions, and they refer patients to secondary and hospital care, acting as gatekeepers. Patients are required to register with a GP for a maximum of 1500 patients for each GP; they can choose their own GP from a list of those available in their area of residence. GPs work for the regional health system as independent professionals and they are paid via a combination of capitation (almost 70%), fee-for-service for specific interventions (ie. vaccination or home care visit) and in some case incentives based on performance (i.e. using ICT).

A recent national health planning legislation (Balduzzi Law No. 189/2012 and the *Patto per la Salute* -Health Pact, 2014–2016) required the Regional Health Systems to re-organise primary care services to promote integration and coordination among GPs and between GPs and other professionals. There were two significant steps in this strategy: 1) the creation of Primary Care Centres (PCCs - in Italy known as “Case della Salute”- ﻿*Health Homes*﻿) and 2) the creation of operational forms that include single professional organisations of GPs, the so-called Functionally Aggregated Groups (FAGs).

The organisational model of PCCs involves the co-location of GPs, who previously mainly worked alone in a single practice, within the same structure with nurses, specialists, social workers and administrative staff. In the Italian PCCs, today it is also possible to have access to other services such as healthcare booking, blood tests, maternal care, vaccinations, and diagnostic imaging. However, this reorganization of primary care delivery has only been partially applied to Italy’s 20 regions [4, 48].

Tuscany is one of the few Regional Health Systems that in Italy have already extensively implemented this organisational model. In fact, the Tuscan Regional Health Authority has invested widely in PCCs in order to provide the population with a unique point of access to primary care services, easily recognisable by population, with the expected effect of reducing the fragmentation of primary care services thereby improving the continuity of care [4, 49].

In 2014, 33 PCCs were active in Tuscany: 8 PCCs had been opened for 5 years or more, while the average age of PCCs was 2.5 years. They involved on average 7 GPs who were co-located mainly with nurses (in 32 PCCs), social workers (in 28 PCCs) and specialists (in 28 PCCs), targeting about 9000 patients on average (from around 2000 to 19,000 patients).

As a result of the second step of primary care re-organisation, in 2014 the Tuscany Regional Health System set up a mono-professional FAG model, aimed at creating local networks of GPs. These FAGs are compulsory networks of GPs: all GPs are required to be member of only one FAG. Each FAG has a coordinator elected by the GPs’ members. The FAGs are replacing all the other kinds of GP association in Tuscany, while in other Italian regions they are not been implemented at all or not so extensively. The FAGs are designed to support local clinical governance and healthcare planning and control, with GPs considered responsible for the continuous improvement of their services’ quality and the safeguard of high care standards. In particular, the FAGs have been promoted with the aim to improve the health system efficiency and quality, focussing on the reduction of unwarranted variation in practice, that is variation in care driven by factors other than population’s health needs and corresponds to the unjustified variability in the evaluation of quality of care indicators within and among the GP networks. Moreover, the FAGs aim to promote the coordination and continuity of care, to facilitate the homogenous definition and implementation of care pathway for chronic patients, to favourite the dissemination of primary care best practices, by using the typical tools of clinical governance, such as audit, peer review, monitoring activities. In Tuscany, all GPs joined one of the 115 FAGs, with an average of 25 GPs per FAG, which then serve a population of around 30,000 patients [50, 51].

### Source of data

The Tuscany Health System carries out periodic patient surveys to monitor the experience and satisfaction with healthcare services (hospitals, emergency departments, maternal care, primary care, etc.) [52–54]. For this study, we used data from a population survey administered at the beginning of 2015 to evaluate patients’ experiences and satisfaction with their GPs [55]. The reference population was the list of adult patients registered with GPs working in Tuscany Region. The survey questionnaire was developed based on the international literature on patient experience measurement and used reporting and rating scales through a 5-point Likert scale, coherently with other international experience on patient surveys [56]. The questionnaire, made up of closed 60 questions, included different sections relating to GP assistance, such as access to GP practise, primary care professionals involved in the practise, communication and relation with the GP, management of chronic conditions, involvement in health promotion activities by the GP and characteristics of patients, in term of socioeconomic condition and health needs.

The survey involved a stratified random sample of patients registered with all the GPs working in the 115 Tuscan FAGs (about 75 patients per FAG stratified by gender and age). The survey reached 8.416 patients, with a response rate of 60%. The sampled patients allowed to estimate the population parameters for each FAG with a confidence level of 95% and a confidence interval of 10%. The survey, conducted by the Management and Health Laboratory of the Sant’Anna ﻿School of Advanced Studies of Pisa, was administered by the Computer Assisted Telephone Interview technique, which was preferred to other data collection techniques because it obtains results quickly and reaches low literacy groups [57]. Due to the aims of our study, in the analysis we considered only FAGs with at least one GP working within a PCC.

### Multilevel analysis

The collected data are hierarchically structured since patient data (level 1) are nested in GPs (level 2), who are in turn nested in FAGs (level 3). Therefore, multilevel linear regression models with random intercept were implemented considering these three levels (patient, GP, FAG) in order to analyse between and within-group variability separately. In fact, patient experiences are expected to vary among GPs and FAGs and to be affected by individual, GP and FAG characteristics. The model allowed identifying the fixed coefficients for the explanatory variables and the random parameters describing the residual unexplained variability in patient satisfaction after taking account of the explanatory variables. This type of statistical model was used to verify both the relationship between patient satisfaction and the co-location of GPs within PCCs when their patients have experience of multidisciplinary team (hypothesis 1) and whether this relationship was stronger for frequent health service users than for the others (hypothesis 2).

To test the second hypothesis that explores the effect modification on the patient satisfaction of co-location of multidisciplinary teams in primary care centres due to the frequent use of health services, the multilevel model was performed on two strata of patient data: frequent user patients and non-frequent user patients. Frequent users of health services were those patients who reported, in the year preceding the survey, that they had made use of at least two different health services (including hospitalisation, admission to an emergency department, or consultation with a specialist) or who had had at least two admissions to at least one of the three services. Non-frequent users of health services were all the other patients, who reported less usage of the three services.

### Outcome and explanatory variables

Patient satisfaction with their GP was used as the key outcome variable to be explained. This was measured as the overall care evaluation, through a 5-point Likert scale. This variable is usually used to evaluate the GP performance within the Tuscany Region healthcare system [50], after being transformed into a 0–100 scale with higher scores indicating better evaluations [57–59]. Specifically ratings of 1, poor; 2, fair; 3, good; 4, very good and 5, excellent were converted to the scores of 0, 25, 50, 75 and 100, respectively, in order to identify more clearly the variability among health services evaluated [57].

The explicative factors of patient satisfaction with the GP introduced in the models can be grouped into:

(i) Patient sociodemographic and health status characteristics and patient usage of GP care, as well as GP characteristics, commonly used to explain the patient level variability in patient surveys; and
(ii) co-location variables, in order to verify our hypotheses.

#### Patient sociodemographic and health status characteristics, patient usage of GP care, and GPs characteristics

We used as covariates of our model: gender, age groups, education, self-reported health status, self-reported chronic conditions, frequency of visits to GP in the previous year, main reason for visits to GP (level 1). GP gender, age and patient practice size were included at level 2.

#### Co-location variables

Co-location was measured by considering both administrative data and patient experience. In relation to administrative data, we used the list of GPs co-located in PCCs, provided by the Regional Health System. In terms of patient experience, we considered the answers of surveyed patients who experienced a GP co-location with a nurse, specialist or other professionals working together to provide a more integrated solution to their needs. The interaction of the above two sources led to the creation of a four-mode variable that identified four subgroups of patients:

- patients assisted by GPs co-located within PCCs, who reported having had an experience of a multidisciplinary team approach (group 1);
- patients of GPs co-located within PCCs, who did not report an experience of a multidisciplinary team approach (group 2);
- patients assisted by GPs not co-located in PCCs, who reported an experience of a multidisciplinary team approach (group 3);
- patients assisted by GPs not co-located in PCCs, who did not report an experience of a multidisciplinary team approach (group 4).

## Results

This study includes data of 2025 patients of GPs belonging to 28 FAGs in Tuscany, where these FAGs have at least one GP working in a PCC. 757 out of 2025 were patients of GPs working in a PCC.

Table 1 describes the characteristics of patients included in this study, grouped according to the co-location variables (see the descriptions of the four groups in the Methods section). The first group consist of patients of GPs within PCCs with experience of multidisciplinary team (9.2% of the sample) and represents the key reference in the following models. The second group consist of patients of GPs within PCCs but without experience of multidisciplinary team (28.1%). The third and fourth groups are made up of patients of GPs not in PCCs, the former with experience of a multidisciplinary team (11.1%) and the latter without any experience of a multidisciplinary team (51.5%). The groups differed significantly only in terms of education, self-reported health status and chronic conditions, as noted in Table 1. Additionally, we observed that patients of GPs working within a PCC had a 65% higher probability of experiencing multidisciplinary team approach than patients of GP not within a PCC (results not in Table).

**Table 1:** Principal characteristics of groups of patients based on co-location

Patient characteristics	Group 1	Group 2	Group 3	Group 4	Total
Gender (%)					
Male	46.3	48.5	42.9	48.6	**47.7**
Female	53.7	51.5	57.1	51.4	**52.3**
Age group (%)					
18–45 y	33.5	37.6	38.8	37.5	**37.3**
46–65 y	33.0	32.2	35.3	34.3	**33.7**
65+ y	33.5	30.2	25.9	28.2	**29.0**
Education (%) (*p* < 0.05) among groups					
Low (primary)	19.6	22.8	14.0	19.8	**20.0**
Medium (secondary)	31.5	28.6	24.6	28.2	**28.2**
High (high school, degree)	48.9	48.6	61.4	52.0	**51.8**
Self-reported health status (%) (*p* < 0.05) among groups					
Bad	17.3	11.2	10.5	11.5	**11.9**
Fair	46.5	51.0	44.1	45.5	**46.9**
Good	36.2	37.8	45.5	43.1	**41.2**
Self-reported chronicity (%) (*p* < 0.05) among groups					
No	61.7	64.3	67.1	71.0	**67.8**
Yes	38.3	35.7	32.9	29.0	**32.2**
Frequency of visits to GP in the last year (%)					
Never	1.1	4.5	5.5	5.0	**4.5**
Rarely (1–4 times/year)	53.8	59.6	55.1	56.3	**56.9**
Sometimes (at least 5 times/year)	45.2	35.9	39.4	38.7	**38.6**
Often (at least once/month)	0.0	0.0	0.0	0.0	**0.0**
Main reason for visit to GP (%)					
Administrative (prescription. certificate)	53.0	52.2	50.9	56.4	**54.3**
Health reason	47.0	47.8	49.1	43.6	**45.7**
Co-location of GP in primary care centres (N. - %)					
Yes	188	569			**37.4**
No			224	1.044	**62.6**
Experience of multidisciplinary team (N. - %)					
Yes	188		224		**20.4**
No		569		1.044	**79.6**
Co-location of multidisciplinary team in primary care centres (N. - %)					
Patients of GP within PCC with experience of multidisciplinary team	188				**9.2**
Patients of GP within PCC without experience of multidisciplinary team		569			**28.1**
Patients of GP not in PCC with other experience of multidisciplinary team			224		**11.1**
Patients of GP not in PCC without any experience of multidisciplinary team				1.044	**51.5**
Satisfaction – overall care evaluation (score) (%) (*p* < 0.05) among groups	87.7	83.7	84.3	83.5	**84.0**

The first group had the greatest proportion of patients reporting a bad health status (17.3%) and chronic conditions (38.3%). The second group had the greatest proportion of patients with a low education (22.8%). Patients in the third group were mainly highly educated (61.4%) and reported a good health status (45.5%); and finally the fourth group had the smallest proportion of patients with chronic conditions (29.0%).

Table 2 reports the patient characteristics by frequent and non-frequent user of health services. The majority of frequent health services users were female (55.8%), reported a bad health status (16.8%) and chronic conditions (43.4%) more often, half had been to a GP at least 5 times during the previous year (49.3%), more often for reasons concerning prescriptions and certificates (56.7%). Patients who did not use health services frequently, on the other hand, were younger (41% in the age group of 18–45 years), had a good health status (50.9%) and no chronic conditions (81.1%). They went to a GP a few times in the previous year (68.0%), but more often for health reasons (48.7%) compared with the other strata (43.3%). There are no statistically significant differences between frequent and non-frequent health services users as to the two key explanatory variables. The proportion of patients of GPs co-located in PCCs were analogous in the two strata, as well as those of patients with experiences of the multidisciplinary team approach.

**Table 2:** Principal characteristics of strata of patients based on frequent use of health services

Patient characteristics	Non frequent HS users (N.927)	Frequent HS users (N.1098)	Total
Gender (%) (*p* < 0.05) among users			
Male	51.9	44.2	**47.7**
Female	48.1	55.8	**52.3**
Age group (%) (*p* < 0.05) among users			
18–45 y	41.0	34.2	**37.3**
46–65 y	33.8	33.6	**33.7**
65+ y	25.2	32.2	**29.0**
Education (%)			
Low (primary)	19.3	20.6	**20.0**
Medium (secondary)	29.9	26.8	**28.2**
High (high school, degree)	50.8	52.6	**51.8**
Self-reported health status (%) (*p* < 0.01) among users			
Bad	6.1	16.8	**11.9**
Fair	43.0	50.2	**46.9**
Good	50.9	33.0	**41.2**
Self-reported chronicity (%) (*p* < 0.01) among users			
No	81.1	56.6	**67.8**
Yes	18.9	43.4	**32.2**
Frequency of visits to GP in the last year (%) (*p* < 0.01) among users			
Never	5.9	3.3	**4.5**
Rarely (1–4 times/year)	68.0	47.4	**56.9**
Sometimes (at least 5 times/year)	26.1	49.3	**38.6**
Often (at least once/month)	0.0	0.0	**0.0**
Main reason for visit to GP (%) (*p* < 0.05) among users			
Administrative (prescription. certificate)	51.4	56.7	**54.3**
Health reason	48.7	43.3	**45.7**
Co-location of GP in primary care centres (%)			
Yes	36.1	38.4	37.4
No	63.9	61.6	62.6
Experience of multidisciplinary team (%)			
Yes	18.9	21.6	**20.4**
No	81.1	78.4	**79.6**
Co-location of multidisciplinary team in primary care centres (%)			
Patients of GP within PCC with experience of multidisciplinary team	8.4	10.0	**9.2**
Patients of GP within PCC without experience of multidisciplinary team	27.7	28.4	**28.1**
Patients of GP not in PCC with other experience of multidisciplinary team	10.5	11.6	**11.1**
Patients of GP not in PCC without any experience of multidisciplinary team	53.4	50.0	**51.5**
Satisfaction – overall care evaluation (score) (%)	84.4	83.7	**84.0**

The patient satisfaction with the GP was generally positive. In a score ranging from 0 to 100, patients rated GP care as 84. The average evaluation scores differed significantly among groups defined on the basis of the co-location of multidisciplinary team in PCCs (Table 1), from 83.5 in the fourth group to 87.7 in the first group. Frequent and non-frequent users of health services (Table 2) assessed the GP care almost equally (respectively 83.7 and 84.4).

### Co-location of multidisciplinary teams in primary care centres

The results of empty model and the likelihood-ratio test indicated the importance of including the FAG and GP level of aggregation (*p* < 0.001), since 7.5% of the total achievement variation is explained by those levels (Table 3). This moderate statistically significant variance observed both at the FAG and GP levels (InCCs respectively 1.2 and 6.3%) is in line with other studies where patient satisfaction variability is mainly explained by patient level [58, 59]. The moderate contextual effect of FAG and GP clusters, which explained respectively only the 1.2 and 6.3% of variance, confirms also the evidence that patient satisfaction is consistently influenced by individual expectations﻿ [60, 61]﻿﻿. The introduction of the patient sociodemographic characteristics variables and the independent variable in the models slightly modify the percentage of variation explained by patient level, while the adjustment for GP characteristics in the full model decreased the ICC at GP level and increased slightly the ICC at FAG level (ICC respectively 1.7 and 5.3%).

**Table 3:** Multilevel model results

Multilevel model results	Null model			Full model		
	FAG = n. 28; GP = n. 549; patient = n. 1.998			FAG = n. 28; GP = n. 538; patient = n. 1.809		
Fixed effects	Coeff.	S.E.	P	Coeff.	S.E.	p
Level 1						
Intercept	83.98	0.45	*p < 0.001*	81.64	5.17	*p < 0.001*
Co-location of multidisciplinary team in primary care centres						
*Patients of GP within PCC with experience of multidisciplinary team (ref)*						
Patients of GP within PCC without experience of multidisciplinary team				-3.65	1.24	*p < 0.01*
Patients of GP not in PCC with other experience of multidisciplinary team				-2.59	1.51	
Patients of GP not in PCC without any experience of multidisciplinary team				-3.44	1.22	*p < 0.01*
Gender						
Female				-0.67	0.66	
Age group *18–45 y (ref)*						
46–65 y				1.38	0.81	
65+ y				1.54	1.08	
Educational level *Low (primary) (ref)*						
Medium (secondary)				-0.52	1.13	
High (high school, degree)				-0.59	1.17	
Self-reported health status *Bad (ref)*						
Fair				3.01	1.13	*p < 0.01*
Good				5.96	1.23	*p < 0.001*
Self-reported chronic conditions						
Yes				1.73	0.78	*p < 0.05*
Frequency of visits to GP in the last year *Never (ref)*						
Rarely (1–4 times/year)				2.69	2.13	
Sometimes (at least 5 times/year)				3.87	2.18	
Main reason for visit to GP Health				2.10	0.67	*p < 0.01*
Level 2						
Gender						
Female				-0.36	0.87	
Age				-0.14	0.07	*p < 0.05*
Patient practice size				0.004	0.001	*p < 0.001*
Random part	Var.	S.E.	CI	Var.	S.E.	CI
Level 3 variance (FAG)	2.43	1.55	0.70–8.47	3.20	1.77	1.08–9.46
Level 2 variance (GP)	10.12	3.97	4.69–21.81	6.93	4.07	2.19–21.91
Level 1 variance (patient)	185.19	6.66	172.58–198.72	182.53	6.99	169.33–196.75
ICC	ICC	S.E.	CI	ICC	S.E.	CI
FAG	0.012	0.008	0.004–0.042	0.017	0.009	0.006–0.048
GP	0.063	0.020	0.034–0116	0.053	0.022	0.023–0.115

The full model results (Table 3) showed that being seen by a GP within a PCC and having the experience of a multidisciplinary team approach were associated with a high satisfaction. In fact, patients of GPs within PCCs without the experience of a multidisciplinary team (group 2) and patients of GPs not in PCCs without any experience of a multidisciplinary team (group 4) were significantly less satisfied compared with patients of a GP co-located in PCC with other professionals and who experienced the joint working of this co-located team (group 1). In addition, patients of a GP not in a PCC with other experiences of a multidisciplinary team were less satisfied (group 3), although the difference with the reference group was not statistically significant.

At the patient level, other statistically significant predictors of patient satisfaction were self-reported health status, self-reported chronic conditions and main reason for visit to GP. Being in fairly good or good health, having chronic conditions and going to the GP for a health reason instead of for a prescription or certificate were positively associated with the patient satisfaction with the GP.

The GP characteristics were slightly associated with patient satisfaction: a negative relationship existed between GP age and satisfaction, and a positive relationship between practice size and satisfaction.

### Frequent health services users

When considering the two strata of patients based on the frequent use of health services, as effect modifier of patient satisfaction, we observed a change in the multilevel model results within the two strata (Table 4). First in the strata of non-frequent health services users, the percentage of variation in patient satisfaction explained by FAG and GP level increased (ICC respectively 4.0 and 9.9%). On the contrary, the FAG and GP cluster are not significant in explaining the variation of patient satisfaction.

**Table 4:** Multilevel model results, by type of users

Stratified multilevel model results	Non frequent health services users			Frequent health services users		
	FAG = n. 28; GP = n. 413; patient = n. 824			FAG = n. 28; GP = n. 456; patient = n. 985		
Fixed part	Coeff.	S.E.	p	Coeff.	S.E.	P
Level 1						
Intercept	83.52	7.49	*p < 0.001*	82.07	7.03	*p < 0.001*
Co-location of multidisciplinary team in primary care centres						
*Patients of GP within PCC with experience of multidisciplinary team (ref)*						
Patients of GP within PCC without experience of multidisciplinary team	-2.63	1.84		-4.50	1.66	*p < 0.01*
Patients of GP not in PCC with other experience of multidisciplinary team	-0.04	2.23		-4.35	1.96	*p < 0.05*
Patients of GP not in PCC without any experience of multidisciplinary team	-1.10	1.81		-5.04	1.59	*p < 0.01*
Gender						
Female	0.34	0.95		-1.43	0.91	
Age group *18–45 y (ref)*						
46–65 y	0.04	1.16		2.65	1.14	*p < 0.05*
65+ y	1.28	1.61		1.63	1.46	
Educational level *Low (primary) (ref)*	-					
Medium (secondary)	0.50	1.65		-0.74	1.54	
High (high school, degree)	-1.10	1.65		-0.55	1.50	
Self-reported health status *Bad (ref)*						
Fair	0.25	2.18		3.86	1.35	*p < 0.01*
Good	2.64	2.24		7.19	1.55	*p < 0.001*
Self-reported chronic conditions Yes	0.51	1.30		2.26	1.00	*p < 0.05*
Frequency of visits to GP in the last year *Never (ref)*						
Rarely (1–4 times/year)	6.43	2.93	*p < 0.05*	-1.96	3.07	
Sometimes (at least 5 times/year)	6.86	3.06	*p < 0.05*	-0.18	3.08	
Main reason for visit to GP Health	1.76	0.95		2.54	0.93	*p < 0.01*
Level 2						
Gender						
Female	0.89	1.24		-1.29	1.14	
Age	-0.25	0.10	*p < 0.05*	-0.06	0.09	
Patient practice size	0.006	0.001	*p < 0.01*	0.004	0.002	*p < 0.05*
Random part	Var.	SE	CI	Var.	SE	CI
Level 3 variance (FAG)	7.38	3.91	2.61–20.87	1.35	2.09	0.07–27.77
Level 2 variance (GP)	10.94	8.72	2.29–52.20	6.41	7.36	0.68–60.77
Level 1 variance (patient)	166.72	11.07	146.38–189.90	187.03	10.81	167.00–209.45
ICC	ICC	SE	CI	ICC	SE	CI
FAG	0.040	0.021	0.014–0.107	0.0067	0.011	0.000–0.127
GP	0.099	0.046	0.039–0.232	0.040	0.037	0.006–0.219

For non frequent health service users, the co-location of a multidisciplinary team in PCCs and patient sociodemographic characteristics were not significantly associated with patient satisfaction. Only having more frequent visits to the own GP in the previous year was associated with a high patient satisfaction.

Conversely, for frequent health service users, the relationships identified in the overall model increased (Table 4). Being seen by a GP working within a PCC and jointly having experienced a multidisciplinary team approach were significantly associated with a better evaluation of the overall care, compared with all the other three groups.

Self-reported health status, self-reported chronic conditions and main reason for visits to GP were significantly associated with patient satisfaction for frequent health service users, with greater coefficients. In this group, patients aged 46–65 years had a higher assessment of the overall care received than younger patients did, while GP characteristics decreased their role in influencing patient satisfaction.

## Discussion

This study shows a strong association between the co-location of GPs in PCCs and the experience of a co-located multidisciplinary team, confirming the findings from previous studies according to which co-location improves joint working [33, 34] and then facilitates multi-professional teamwork [20, 21].

However, our first hypothesis was that co-location in itself would not be sufficient to have positive relationship with patient experience. Our key interest was thus to investigate the interaction between the co-location of GPs in PCCs and the experience of a multidisciplinary team approach. Our findings show that the co-location and the experience of a multidisciplinary team in PCCs (as the interaction between the two variables deriving from patient experience data and administrative data) were significantly associated with patient satisfaction. In fact, patients (group 1) were more satisfied when their GPs were co-located with other health and social professionals in PCCs and they perceived that co-located professionals were working in collaboration.

Compared with these patients, a lower satisfaction was found by those patients who had been seen by GPs working within PCCs, but who had not experienced a multidisciplinary team (group 2). This result can be explained in different ways. The GPs of these patients probably still worked both in a PCC and in a single practice [4] and the interviewed patients went to see their GP outside the PCC. Otherwise the GPs and other professionals co-located in PCCs continued to provide healthcare as separate silos [30, 31]. In both cases, patients missed the benefits of joint work with co-located professionals and this was negatively associated with patient satisfaction.

Patients of GPs not working in PCCs with experience of a multidisciplinary team approach (group 3) were less satisfied than those patients with an analogous experience and who had been seen by GPs in PCCs, however this difference was not statistically significant. This seems to suggest again that what is more important for patients are the benefits they perceive that they have received from the collaboration and cooperation among professionals, which are key aspects of a multidisciplinary team [8], irrespective of the specific setting where they are co-located - PCCs, polyclinics, or other types of organisations [62, 63].

In addition, co-locating GPs and other professionals in PCCs is strongly highlighted when focusing on frequent health services users. These patients are older, with a bad health status and more chronic conditions; they frequently go to GPs and have had more referrals to hospitals, emergency department or specialists. This profile describes patients with complex needs, who have an intense contact with health care services and require a complex management of care involving multiple clinicians in different care settings [9, 64]. For these patients, being seen by GPs working in PCCs and implementing a multidisciplinary team approach may lead to a higher satisfaction compared with all the other options analysed. However, we are aware that our analysis does not produce evidence of a cause-effect relationship between co-location and satisfaction, but only highlights whether an association exists between them.

The lowest level of satisfaction of frequent health services users was associated with being seen by a GP not working in a PCC and not having any experience of a multidisciplinary team. These findings confirm our second hypothesis that the co-location of multiprofessional teams in PCCs is a predictor of higher patient satisfaction for frequent healthcare services. The co-location of GPs with other professionals and their joint working as experienced in PCCs seems to represent a greater benefit for patients with complex needs who frequently use primary care, hospital, emergency care and specialized care. These patients have a greater risk of fragmentation in their care pathway [19], and they require a more coordinated and comprehensive care [10]. The solution they receive by GPs co-located in PCCs seems to address their needs better. This may be due to the more effective collaboration of professionals within PCCs in managing these patients, in line with the evidence that co-location in these settings particularly facilitates collaboration and cohesion among professionals [65]. Indeed, co-location helps in the effective use of time and resources, facilitates communication and information sharing, and improves relationships between professionals and between professionals and patients [66, 67].

The co-location of primary care teams, with a single point of access, contributes to reducing duplication and to ensuring services that are more responsive for patients [68]. It may also be due to a greater capacity of PCCs to provide a broad, specialized and preventive care for people with chronic diseases, thanks to their organisational characteristics. These characteristics may be the increased delivery of disease management programmes, the increased involvement of nurses in service provisions for patients with complex and chronic conditions, or the increased availability of equipment in a practice that facilitate the early management of deteriorating patients and reduce unnecessary referrals [69]. Finally, PCCs help patients with complex needs more effectively, probably because they manage their transitions between providers more appropriately, thanks to care co-ordination mechanisms and shared procedures for referral with secondary care [63], which help patients to perceive more continuity and coherence in their care pathway.

The results regarding the higher satisfaction of frequent users of health services with GPs working in PCCs could be valuable for the scientific and professional communities because they add information about the level of satisfaction of more complex patients. In fact, PCCs seem particularly able to handle the demands of complex patients, who usually report a high level of dissatisfaction explained, among other reasons, by a rising complexity of health problems [70].

One strength of this study is to provide evidence of a positive association between co-location in PCCs and patient satisfaction when patients themselves were also aware of the existence of this co-location. The study also used data from a large population based survey, with a consistent response rate, aimed at evaluating patient experiences of GP healthcare [55]. The survey sample was representative at the FAG level, which is the smallest level of administrative grouping of GP patients, and which made it possible to distinguish between the variability in patients, GPs and FAG levels of patient satisfaction. Although the results cannot be generalised to other contexts, Tuscany is one of the largest regions in Italy where co-location in PCCs has mostly been implemented and is highly developed [4, 48, 49]. This study is easily replicable in other contexts with experiences of co-located GPs with other professionals in PCCs.

### Study limitations

The associations we found between patient satisfaction and co-location of GPs in PCCs cannot be interpreted causally, although it is likely that organisational factors may have an influence on patient outcome. Information was lacking concerning the other types of organisational models where GPs were involved, such as single practices, polyclinics or other settings, in order to verify whether differences in patient satisfaction occurred - in any case, this was not within the scope of this study.

Although the constitution of PCCs is a national and regional strategy addressing all GPs, a potential bias in our study can be related to the selection of GPs within PCCs. GPs might have chosen to work in PCCs because they considered relevant the integrated care approach and had already a positive attitude to inter-professional collaboration within a multidisciplinary team. However, the co-location of GPs in PCCs is not only bottom up approach due to the GPs’ individual choice, but also a top down approach because the Regional health care system and Local Health Authorities are facilitating and encouraging GPs to work in a PCCS.

Moreover, it is not taken for granted that GPs co-located in a PCC have a positive attitude to multidisciplinary approach, because they can still work separately from the other professionals working in the same setting, as some literature pointed out [30], while there can be multidisciplinary teams operating also in other settings different from PCCs. There is not selection bias risk for patients since they do not choose personally to go to PCCs, but they use the PCC services if their GPs work there. In this sense, GPs in PCCs have more patients reporting chronic conditions not because they end up there according to their status, while they may be more aware of their conditions due to a more proactive care received by GPs in PCCs.

Data concerning the involvement of GPs in disease management programmes might improve the analysis and produce additional evidence on the studied relationships, and the authors intend to collect new data and conduct new research in this direction in the near future.

## Conclusions

This article has highlighted a positive relationship between the co-location of a multidisciplinary team in PCCs and patient satisfaction with their GPs, especially for frequent health service users. Co-location produces a positive patient perception of care, probably because co-location facilitates the joint working among different professionals, although we are aware that colocation is not sufficient to ensure always a multidisciplinary teamwork.

In order to improve patient satisfaction, health and social professionals co-located in PCCs should be supported in order to change their way of working. This should be done taking into account that patients are more satisfied when they know that more than one professional is available in the structure to provide a more integrated solution to their needs. This should lead professionals to actually collaborate as a multidisciplinary team. However, this is a long-term task requiring training, considering that many health professionals are not been trained to work inter-professionally or have had no experience of doing so [69]. In addition, shared information systems, governance changes with clear objectives, and above all constant adjustments are important to strengthen care coordination [71].

The policy makers have to consider that the co-location of a multidisciplinary team in a PCC does not involve just relocating care based on physical proximity, but above all redesigning it with the aim of integrated care [29]. It is not relevant to promote the co-location of GPs with other professionals without promoting in the same time professional integration and the constitution of multidisciplinary teams who are effectively recognised and appreciated by patients. Indeed, the higher satisfaction is related to both conditions (1. GPs that work in a PCC and 2. Patients that perceived the multidisciplinary experience of care). This positive experience of patients, who perceive a real change in the way they are assisted, represents the surplus value that should be achieved by PCCs. Furthermore, the implementation of the PCCs model requires both “hard skills” related to the co-location needs (i.e. the place and the structures, ICT system…), and “soft skills” related to the non-technical competences of involved professionals (i.e. inter-professional background, communication …).

Complex patients who use frequently health services have already acknowledged this surplus value of PCCs. Therefore, the co-location of a multidisciplinary team in a PCC should be promoted as the most appropriate organisational model to improve the care experience of patients with complex health and social needs. In particular, health authorities of territories with large fragile population should be the most active in the implementation of the PCC model and in the involvement of all GPs in this co-located setting.

## Abbreviations

FAG: Functionally aggregated group
GP: General practitioner
PCC: Primary care centre

## References

[1] Bourgueil Y, Marek A, Mousquès J (2009). Three models of primary care organisation in Europe, Canada, Australia and New-Zealand. Questions d’économie de la santé.

[2] Kokko S (2009). Integrated primary health care: Finnish solutions and experiences. Int J Integr Care.

[3] Afrite A, Bourgueil Y, Daniel F, Mousquèsa F, Couralet PE, Chevillard G (2013). The impact of multi-professional group practices on healthcare supply. Evaluation aims and methods for “maisons”, “pôles de santé” and “centres de santé” within the framework of experiments with new mechanisms of remuneration. Questions d’économie de la santé.

[4] Bonciani M, Barsanti S, Matarrese D (2015). Esperienze di integrazione nell’assistenza primaria basate sulla co-location dei servizi: quali prospettive per il modello della Casa della Salute?. Mecosan.

[5] Campbell H, Hotchkiss R, Bradshaw N, Porteous M (1998). Critical care pathways. Br Med J.

[6] Hern T, Talen M, Babiuch C, Durazo-Arvizu R (2009). Patient care management teams: improving continuity, office efficiency, and teamwork in a residency clinic. J Grad Med Educ.

[7] OECD. Doing Better for Families. OECD Publishing; 2011. ISBN 978-92-64-09872-5.

[8] Øvretveit J (1993). Coordinating community care: multidisciplinary teams and care management.

[9] Carter S, Garside P, Black A (2003). Multidisciplinary team working, clinical networks, and chambers; opportunities to work differently in the NHS. Qual Saf Health Care.

[10] Mitchell GK, Tieman JJ, Shelby-James TM (2008). Multidisciplinary care planning and teamwork in primary care. MJA.

[11] Tanaka M (2003). Multidisciplinary team approach. Geriatr Gerontol Int.

[12] Wagner EH (2000). The role of patient care teams in chronic disease management. BMJ.

[13] Codispoti C, Douglas MR, McCallister T, Zuniga A (2004). The use of a multidisciplinary team care approach to improve glycemic control and quality of life by the prevention of complications among diabetic patients. J Okla State Med Assoc.

[14] Liberman RP, Hilty D, Drake R, Tsang H (2001). Requirements for multidisciplinary teamwork in psychiatric rehabilitation. Psychiatr Serv.

[15] Taylor C, Munro SJ, Glynne-Jones R, Griffith C, Trevatt P, Richards M, Ramirez AM (2010). Multidisciplinary team working in cancer: what is the evidence?. BMJ.

[16] Taylor C, Finnegan-John J, Green JSA (2014). “No decision about me without me” in the context of cancer multidisciplinary team meetings: a qualitative interview study. BMC Health Serv Res.

[17] Lamb BW, Jalil RZ, Sevdalis n, Vincent C, Green JSA (2014). Strategies to improve the efficiency and utility of multidisciplinary team meetings in urology cancer care: a survey study. BMC Health Serv Res.

[18] Clarke DJ (2013). The role of multidisciplinary team care in stroke rehabilitation. Prog Neurol Psychiatry.

[19] Maneze D, Dennis S, Chen HY, Taggart J, Vagholkar S, Bunker J, Liaw ST (2014). Multidisciplinary care: experience of patients with complex needs. Aust J Prim Health.

[20] Coburn AF (2001). Models for integrating and managing acute and long-term care services in rural areas. J Appl Gerontol.

[21] Suter E, Oelke ND, Adair CE, Armitage GD (2009). Ten Key principles for successful health systems integration. Healthc Q (Toronto, Ont).

[22] Watson D, Townsley R, Abbot D (2002). Exploring multi-agency working in services to disabled children with complex healthcare needs and their families. J Clin Nurs.

[23] Holtom M (2001). The partnership imperative: joint working between social services and health. J Manag Med.

[24] Hubbard G, Themessl–Huber M (2005). Professional perceptions of joint working in primary care and social care services for older people in Scotland. J Interprof Care.

[25] Hudson B (2006). Integrated team working: You can get it if you really want it: part 1. J Integrated Care.

[26] Blount A (2003). Integrated Primary Care: Organizing the Evidence. Fam Syst Health.

[27] Collins C, Hewson DL, Munger R, Wade T (2010). Evolving models of behavioral health integration in primary care.

[28] Cameron A, Lart R, Bostock L, Coomber C (2014). Factors that promote and hinder joint and integrated working between health and social care services: a review of research literature. Health Soc Care Community.

[29] Imison C, Naylor C, Maybin J (2008). Under One roof: will polyclinics deliver integrated care?.

[30] Lawn S, Lloyd A, King A, Sweet L, Gum L (2014). Integration of primary health services: being put together does not mean they will work together. BMC Res Notes.

[31] Davey B, Levin E, Iliffe S, Kharicha K (2005). Integrating health and social care: implications for joint working and community care outcomes for older people. J Interprof Care.

[32] Ginsburg S (2008). Colocating health services: a way to improve coordination of children’s health care?. Commonwealth Fund pub.

[33] Gibb C, Morrow M, Clarke C, Cook G, Gertig P, Ramprogus V (2002). Transdisciplinary working: evaluating the development of health and social care provision in mental health. J Ment Health.

[34] Frost N. Professionalism, partnership and joined-up thinking: a research review of front-line working with children and families. Research in practice. 2005. ISBN 0-904984-04-5

[35] Vuori H (1987). Patient satisfaction-an attribute or indicator of the quality of care?. QRB Qual Rev Bull.

[36] Donabedian A (1988). The quality of CareHow Can It Be assessed?. JAMA.

[37] Lewis JR (1995). Patient views on quality care in general practice: literature review. Soc Sci Med.

[38] Jaques H (2012). Putting patients at the heart of quality. BMJ.

[39] Baker R, Streatfield J (1995). What type of general practice do patients prefer? exploration of practice characteristics influencing patient satisfaction. Br J Gen Pract.

[40] Baker R (1996). Characteristics of practices, general practitioners and patients related to levels of patients’satisfaction with consultation. Br J Gen Pract.

[41] Branson C, Badger B, Dobbs F (2003). Patient satisfaction with skill mix in primary care: a review of the literature. Prim Health Care Res Dev.

[42] Jung HP, Baerveldt C, Olesen F, Grol R, Wensing M (2003). Patient charactristics as predictors of primary health care preferences: a systematic literature analysis. Health Expect.

[43] Haddad S, Potvin L, Roberge D, Pineault R, Remondin M (2000). Patient perception of quality following a visit to a doctor in a primary care unit. Fam Pract.

[44] Berchtold P, Kunzi B, Busato A (2011). Differences of the quality of care experience: the perception of patients with either network or conventional health plans. Fam Pract.

[45] Grol R, Wensing M, Mainz J, Jung HP, Ferreira P, Hearnshaw H (2000). Patients in Europe evaluate general practice care: an international comparison. Br J Gen Pract.

[46] Kroneman MW, Maarse H, van der Zee J (2006). Direct access in primary care and patient satisfaction: a European study. Health Policy.

[47] Schäfer WLA, Boerma WGW, Murante AM, Sixma HJM, Schellevis FG, Groenewegen PP (2015). Assessing the potential for improvement of primary care in 34 countries: a cross-sectional survey. Bull World Health Organ.

[48] Odone A, Saccani E, Chiesa V, Brambilla A, Brianti E, Fabi M, Curcetti C, Donatini A, Balestrino A, Lombardi M, Rossi G, Saccenti E, Signorelli C (2016). The implementation of a Community Health Centre-based primary care model in Italy. The experience of the Case della Salute in the Emilia-Romagna Region. Ann Ist Super Sanita.

[49] Barsanti S, Bonciani M, Roti L. Il Quaderno delle Case della Salute. Firenze, 2016 Edizioni Polistampa ISBN 978-88-596-1625-2

[50] Barsanti S, Nuti S. Il Sistema di valutazione della performance delle AFT toscane. Report 2014. Pisa, 2015 Edizioni ETS ISBN 978-88-8250-162-4

[51] Barsanti S, Bonciani M, Vola F, Pirisi L (2016). Innovatori, indecisi, bisognosi o autonomi. I medici di medicina generale tra integrazione e accountability. Mecosan.

[52] Murante AM, Vainieri M, Rojas DC, Nuti S (2014). Does feedback influence patient - professional communication? Empirical evidence from Italy. Health Policy.

[53] De Rosis S, Barsanti S (2016). Patient satisfaction, e-health and the evolution of the patient-general practitioner relationship: Evidence from an Italian survey. Health Policy.

[54] Nuti S, Murante AM, Matarrese D. Report of the maternity pathway. Edizioni Polistampa. 2015. ISBN 978-88-596-1571-2

[55] Murante AM (2015). Indagine sull’esperienza degli utenti della medicina generale 2014-2015.

[56] Coulter A, Fitzpatrick R, Cornowell J (2009). The Point of Care. Measures of Patients’ Experience in Hospital: Purpose, Methods and Uses.

[57] Brown AD, Sandoval GA, Murray M (2008). Comparing patient reports about hospital care across a Canadian–US border. Int J Qual Health Care.

[58] Murante AM, Seghieri C, Brown S, Nuti S (2014). How do hospitalization experience and institutional characteristics in fluence inpatient satisfac tion? A multilevel approach. Int J Health Plann Mgmt.

[59] Sjetne IS, Veenstra M, Stavem K (2007). The effect of hospital size and teaching status on patient experiences with hospital care: a multilevel analysis. Med Care.

[60] Sixma HJ, Spreeuwenberg PM, van der Pasch MA (1998). Patient satisfaction with the general practitioner: a two-level analysis. Med Care.

[61] Berhane A, Enquselassie F (2016). Patient expectations and their satisfaction in the context of public hospitals. Patient Prefer Adherence.

[62] Wen J, Schulman K (2014). Can team-based care improve patient satisfaction? a systematic review of randomized controlled trials. PLoS One.

[63] Sheaff R, Halliday J, Øvretveit J, Byng R, Exworthy M, Peckham S, Asthana S. Integration and continuity of primary care: polyclinics and alternatives – a patient-centred analysis of how organisation constrains care co-ordination. Health Services and Delivery Research. 2015;3(35). doi:10.3310/hsdr03350. 26312365

[64] Schoen C, Osborn R, How S, Doty M, Peugh J (2009). In chronic condition: experiences of patients with complex health care needs, in eight countries, 2008. Health Aff.

[65] Oandasan IF, Gotlib Conn L, Lingard L, Karim A, Jakubovicz D, Whitehead C, Miller K-L, Kennie N, Reeves S (2009). The impact of space and time on interprofessional teamwork in Canadian primary health care settings: implications for health care reform. Prim Health Care Res Dev.

[66] Cameron A, Lart R (2012). Revisiting joint working. J Integrated Care.

[67] Ham C. Only connect. Policy options for integrating health and social care. The Nuffield Trust. 2009.

[68] Rumball-Smith J, Wodchis WP, Koné A, Kenealy T, Barnsley J, Ashton T (2014). Under the same roof: co-location of practitioners within primary care is associated with specialized chronic care management. BMC Fam Pract.

[69] Poot AJ, Wendy P. J. den Elzen, Jeanet W. Blom, Jacobijn Gussekloo. Level of Satisfaction of Older Persons with Their General Practitioner and Practice: Role of Complexity of Health Problems. Plos One. 2014;9(4):e94326. doi:10.1371/journal.pone.0094326. 10.1371/journal.pone.0094326 PMC3978057 24710557

[70] Sloper P (2004). Factors and barriers for co-ordinated multi agency services. Child Care Health Dev.

[71] Van Houdt S, Heyrman J, Vanhaecht K, Sermeus W, De Lepeleire J (2013). Care pathways across the primary-hospital care continuum: using the multi-level framework in explaining care coordination. BMC Health Serv Res.

